# Mediation CNN (Med-CNN) Model for High-Dimensional Mediation Data

**DOI:** 10.3390/ijms26051819

**Published:** 2025-02-20

**Authors:** Yao Li, Zhongyuan (Jasper) Zhang, Olli Saarela, Divya Sharma, Wei Xu

**Affiliations:** 1 Dalla Lana School of Public Health, University of Toronto, Toronto, ON M5S 1A1, Canada; yaojenny.li@mail.utoronto.ca (Y.L.); jasperz.zhang@mail.utoronto.ca (Z.Z.); olli.saarela@utoronto.ca (O.S.); divya03@yorku.ca (D.S.); 2 Princess Margaret Cancer Centre, University Health Network, Toronto, ON M5G 2C1, Canada; 3 Department of Mathematics and Statistics, York University, Toronto, ON M3J 1P3, Canada

**Keywords:** high-dimensional, mediation analysis, microbiome, deep learning

## Abstract

Complex biological features such as the human microbiome and gene expressions play a crucial role in human health by mediating various biomedical processes that influence disease progression, such as immune responses and metabolic processes. Understanding these mediation roles is essential for gaining insights into disease pathogenesis and improving treatment outcomes. However, analyzing such high-dimensional mediation features presents challenges due to their inherent structural and correlations, such as the hierarchical taxonomic structures in microbial operational taxonomic units (OTUs), gene–pathway relationships, and the high dimensionality of the datasets, which complicates mediation analysis. We propose the Med-CNN model, an iterative approach using Convolutional Neural Networks (CNNs) to incorporate the complex biological network of the mediation features. The output values from network-specific CNN models are condensed into an integrative mediation metric (IMM), which captures essential biological information for estimating mediation effects. Our approach is designed to handle high-dimensional data and accommodate their unique structures and non-linear interactive mediation effects. Through comprehensive simulation studies, we evaluated the performance of our algorithm across different scenarios, including various mediation effects, effect sizes, and sample sizes, and we compared it to conventional methods. Our simulations demonstrated consistently lower biases in mediation effect estimates, with values ranging from 0.17 to 0.56, which were lower than other established methods ranging from 0.24 to 13.27. In a real data application, our method identified a mediation effect of 0.06 between ethnicity and vaginal pH levels.

## 1. Introduction

Mediation analysis is widely applied in biomedical research to understand how treatments impact outcomes through various biological features. Baron and Kenny first introduced the concept of mediation within the structural equation modeling framework in their 1986 paper [1]. Another widely adopted approach is causal mediation analysis within the counterfactual framework. Both approaches have undergone substantial development, enabling the handling of low-dimensional [2,3,4,5,6,7] and high-dimensional mediators [8,9,10,11,12].

The advancement of next-generation sequencing technology has deeply impacted the scope of available biological data, which has enabled detailed profiling of biological datasets such as microbial communities and genetic variations. This progress has resulted in complex, high-dimensional datasets, such as a vast number of operational taxonomic units (OTUs) in microbiome studies and comprehensive gene expression profiles in genetic research. These datasets raise unique challenges for mediation analysis due to their high dimensionality, non-linear interactive effects between features, and complex inherent structures, such as the hierarchical taxonomic structure in the microbiome and gene–pathway relationships.

Many studies have highlighted the roles of the human microbiome and gene expression as potential mediators in the path from clinical exposures to disease outcomes [13,14,15,16,17]. The composition of the human microbiome is dynamic and influenced by many factors, such as diet and medication, refs. [18,19,20,21], and it is associated with several diseases, including obesity, type 2 diabetes, and cardiovascular disease [22,23,24]. Similarly, gene expression plays a critical role in understanding disease mechanisms, as the expression of genes influences disease outcomes by regulating biological processes such as immune responses and metabolism [25,26].

A key challenge in analyzing both microbiome and gene expression as mediators arises from their hierarchical structures and the complex interactive relationships among features. In the microbiome, OTUs within the same taxonomic group often interact and function collaboratively, while OTUs from different taxa may function more independently. These relationships often involve non-linear interactions, which add complexity to accurately estimating mediation effects. Similarly, gene expression data present gene pathway structures, where genes within the same pathway are often correlated and work coordinately. These structural relationships, combined with potential non-linear interactions, add complexity to mediation analysis.

Most existing high-dimensional mediation methods primarily focus on dimension reduction but do not explicitly capture complex interactive mediation effects. For example, Zhang et al. employed a regularization-based method to assess the mediation effects of DNA methylation markers between environmental exposures and health outcomes [10]. Similarly, Zhao et al. introduced the pathway LASSO for analyzing genetic and brain mediators [11]. Chén et al. developed the directions of the mediation method for neuroimaging data [12]. Huang and Pan proposed a principle component analysis-based approach to transform high-dimensional gene expression mediators into conditionally independent marginal mediation models to analyze the genomic contribution to patient survival in glioblastoma multiforme [27]. Although these methods provide useful tools for high-dimensional mediation analysis, they often fail to capture the complex interactions and hierarchical structures inherent in biological datasets, which are critical for understanding mediation effects comprehensively.

In contrast, CNNs naturally capture complex interactions and hierarchical structures and have been widely adopted to analyze high-dimensional biomedical data. For example, the ReGeNNe method employs CNNs to incorporate the biological clustering of genes within pathways and capture interactions between pathways for disease prediction [28]. Similarly, the TaxoNN applies CNNs to account for inherent correlations and non-linear relationships in microbiome data to enhance disease prediction accuracy [29].

To leverage these strengths for causal mediation, we introduce Med-CNN, a novel high-dimensional causal mediation model that employs CNNs to transform high-dimensional biological data into an integrative mediation metric (IMM) [29,30]. By synthesizing features within networks, Med-CNN derives network-specific values, which are then concatenated to predict an IMM. This approach captures the aggregate impact of the network in mediating the treatment-to-outcome relationship and effectively addresses the complexities of high-dimensional mediation analysis. Our method combines the predictive power of deep learning with the theoretical framework of mediation analysis, which allows Med-CNN to learn the inherent data structures among complex biological datasets.

## 2. Results

### 2.1. Simulation Results

In the first simulation, we evaluated the performance of Med-CNN under both null and alternative mediation effect scenarios. The results are shown in Table 1. Across all scenarios, our approach showed consistently low bias and standard deviation (SD) for both the natural indirect effect (NIE) and natural direct effect (NDE). In the null scenarios (Scenarios 1–3), where there was no mediation effect, the bias for NIE ranged from −0.08 to −0.002, and the NDE ranged from 0.04 to 0.09. The SD values for the NIE were between 0.11 and 1.15, while for the NDE, they ranged from 0.18 to 1.15. In particular, Scenario 1 ($\alpha_{2}=\beta_{1}=0$) showed the smallest SD, as the absence of both effects reduced the complexity of the parameter estimation. In contrast, Scenario 2 ($\alpha_{2}=0$, $\beta_{1}\neq 0$) had a larger SD compared to Scenario 3 ($\beta_{1}=0$, $\alpha_{2}\neq 0$). This is due to the fact that in Scenario 2, $\beta_{1}\neq 0$ introduced multicollinearity between IMM and *A*, leading to greater variability in the estimated coefficients. In the alternative scenario (Scenario 4), where mediation effects were present, we examined the model’s performance across a range of $\beta_{1}$ values from −5 to 5. For the NIE, the bias ranged from −0.22 to 0.56, with SD values between 0.68 and 1.74. For the NDE, the bias remained within −0.58 to 0.20, with the SD values ranging from 0.24 to 1.47.

In Simulation 2, we compared the performance of Med-CNN with the spectral decomposition-based method (SD-based) and the regularization-based approach (Reg-based) under the non-zero mediation effect scenario [10,27]. The results are summarized in Table 2. In the linear feature–network setting, the SD-based method achieved the lowest bias, with values of 0.24 for the NIE and −0.11 for the NDE and SDs of 0.75 and 0.29, respectively. These results indicate that the SD-based method performs well in linear conditions due to its ability to transform and simplify the mediators into a set of linear combinations of features that explain most of the variability, which aligns closely with the structure of the data. The Med-CNN showed moderate estimation accuracy with a bias of 0.56 for the NIE and −0.58 for the NDE, with SDs of 1.74 and 1.47, respectively. While it did not outperform the SD-based method, it still achieved better results compared to the Reg-based method. In the non-linear feature–network setting, Med-CNN had the lowest bias and SD, with a bias of 0.17 for the NIE and −0.19 for the NDE. These results demonstrate Med-CNN’s efficiency in estimating mediation effects under non-linear conditions compared to both SD-based and Reg-based methods.

In the third simulation (Table 3), we examined the impact of feature interactions on the performance of our approach in comparison to the SD-based and Reg-based methods. In the linear feature–network setting, Med-CNN achieved the highest accuracy with a bias of 0.42 for the NIE and −0.44 for the NDE, with SDs of 0.81 and 0.46, respectively. While the SD-based method had slightly lower SDs (0.72 for the NIE and 0.86 for the NDE), its much larger bias (6.68 for the NIE and −6.54 for the NDE) makes it less accurate. The Reg-based method showed both higher bias (12.82 for the NIE and −12.05 for the NDE) and higher SDs (5.20 for the NIE and 5.10 for the NDE). These results suggest that Med-CNN has a better balance between bias and variability in estimating mediation effects under linear conditions. In the non-linear feature–network setting, Med-CNN maintained low bias values (0.27 for the NIE and −0.29 for the NDE) and SDs (0.75 for the NIE and 0.39 for the NDE), which outperformed the other two methods in accounting for interactions. The SD-based method had large bias values (13.27 for the NIE and −13.14 for the NDE) under this non-linear condition, suggesting that it has difficulty handling complex feature interactions due to its design for linear relationships. The Reg-based method, however, showed improved performance, with a bias of 0.81 for the NIE and −0.76 for the NDE.

In the fourth simulation, we evaluated convergence thresholds of 1%, 0.1%, and 0.01% to determine the optimal setting for Med-CNN training under scenarios with a non-zero NIE. Table 4 presents the results, showing that both the 0.1% and 0.01% thresholds demonstrated similar performance in terms of bias and SD for the NIE and the NDE estimates. However, the 0.01% threshold produced the lowest bias and SD values, which suggests slightly better accuracy. Although the 0.1% threshold may require fewer iterations, we recommend the 0.01% threshold as the optimal convergence criterion for our model due to its better accuracy in parameter estimation.

In the fifth simulation, we assessed scalability by evaluating Med-CNN’s performance across sample sizes of 500, 1000 and 1500. The results, presented in Table 5, showed that as the sample size increased, Med-CNN demonstrated reduced bias and smaller SD values, highlighting its improved stability and performance for large-scale datasets.

### 2.2. Real Data Analysis Results

**Figure 1:**
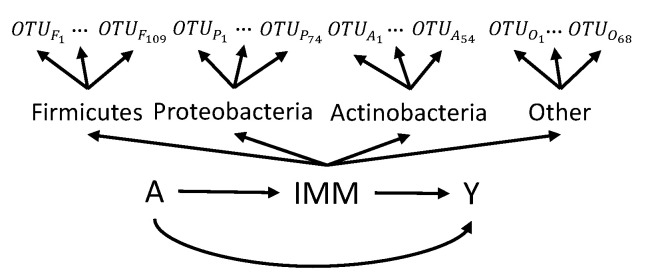
The DAG of the real-data example. *A* is ethnicity, IMM is the predicted integrative mediation metric, *Y* is the vaginal pH level.

The results of the real data analysis and the graphical illustration are shown in Table 6 and Figure 1. We found that the estimated NIE came out to 0.06, indicating that the vaginal microbiome mediates 24% of the effect from the exposure to the outcome.

Previous studies have established that the relative abundance of some of the vaginal microbiome varies among different ethnic groups and could be associated with vaginal pH levels [31,32,33,34,35]. Our results align with the existing studies, supporting the potential role of the microbiome concerning ethnicity and vaginal pH levels. By quantifying the mediation effect of the entire set of vaginal OTUs on pH levels, our analysis provides a more comprehensive understanding of the microbiome’s role in this context.

## 3. Discussion

In this study, we introduced Med-CNN, a deep-learning approach for estimating mediation effects within high-dimensional biological data. Our novel approach simplified the process of estimating the mediation effects in complex biological structures by effectively integrating multiple networks into an integrative mediation metric while accounting for the interactive effects among biological features within these networks. Through comprehensive simulation studies, we demonstrated that Med-CNN produces accurate and stable estimates of the NIE and NDE across varying effect sizes, sample sizes, and complexity levels, validating its reliability in different scenarios. Additionally, our real-data application illustrated Med-CNN’s ability to capture the overall mediation effect of the microbiome while accounting for hierarchical and interactive relationships among OTUs. Unlike traditional methods that often focus on individual OTUs and ignore the complex hierarchical structure and interactions within the microbiome, Med-CNN provides a comprehensive view of how OTUs collectively mediate the relationship between ethnicity and vaginal pH levels.

Our simulations provided additional insights into Med-CNN’s performance under different null hypothesis scenarios. We observed a fluctuation in the SDs of the NIE estimates ranging from 0.11 to 1.15. This variability highlights how the setup of the causal mediation model can either inflate or reduce the variability in mediation effect estimates. For example, in the first null hypothesis scenario ($\alpha_{2}=\beta_{1}=0$), where both the exposure-to-mediator and mediator-to-outcome pathways were disrupted, the parameter space was simplified, resulting in a smaller SD. In contrast, when only the mediator-to-outcome pathway was zero ($\alpha_{2}=0$), the multicollinearity between IMM and *A* resulted in greater variability compared to the third scenario ($\beta_{1}=0$), where an exposure-to-mediator pathway was absent.

Med-CNN demonstrated clear advantages over SD-based and Reg-based approaches, particularly in scenarios with non-linear relationships or feature interactions within the network. In simpler cases with no interactions and purely linear relationships among features, the SD-based method slightly outperformed Med-CNN in terms of bias and SD estimation. This is likely due to the alignment of the SD-based architecture with linear data structures, which enables it to reduce dimensionality efficiently while capturing most of the variability. Nevertheless, Med-CNN consistently outperformed the Reg-based approach and excelled in scenarios with non-linearity or complex interactions. This highlights Med-CNN’s strength in handling the complexities of high-dimensional biological datasets.

Our study also has limitations that should be addressed in future work. The current causal mediation model focused on accurately estimating the mediation effects; however, we did not incorporate confounders or conduct formal hypothesis testing with p-values and confidence intervals to quantitatively assess the statistical validity of our results. Incorporating confounders into the Med-CNN is feasible, but it presents challenges related to model convergence and computational complexity. Confidence intervals can be approximated using standard errors (SE) derived from the delta method (details in Appendix D) under the assumption that IMM is known, but this assumption may underestimate the SE of NIE. Alternatively, bootstrap procedures could be employed for empirical confidence intervals. However, developing stringent hypothesis testing for Med-CNN requires further methodology development, particularly in exploring the asymptotic properties of the estimates. In addition, we did not include a feature importance mechanism to identify specific mediators driving the mediation effect, as our primary objective was to estimate the overall mediation effect. Incorporating feature importance analysis could provide deeper biological insights and improve the interpretability of results.

Although Med-CNN handles data sparsity and noise through normalization during preprocessing, missing data remains a common challenge in real-world studies. Future efforts could focus on integrating automated handling of missing data directly into the model pipeline. This would further enhance the applicability of Med-CNN in practical settings.

Despite these limitations, Med-CNN is an innovative deep-learning approach in high-dimensional mediation analysis. By leveraging convolutional layers, it is well suited for large-scale datasets with complex biological structures, including gene–pathway relationships in gene expression studies, voxel-level spatial dependencies in neuroimaging, and hierarchical correlations in microbiome research. For instance, Med-CNN could model causal mediation in gene expression data by treating pathways as structured networks of genes, capturing the interactions within the pathways. This novel approach not only captures the non-linear interactive effects between the biological features but also effectively integrates their inherent structural complexities. Future directions will focus on integrating confounders, implementing hypothesis testing frameworks, refining the architecture to incorporate feature importance analysis, and improving the model’s adaptability to practical challenges. As a deep-learning model that bridges statistical mediation models with high-dimensional biological research, Med-CNN provides a powerful tool to advance the understanding of mediation mechanisms in complex biological studies.

## 4. Materials and Methods

### 4.1. Review of Natural Direct and Indirect Effects

**Figure 2:**
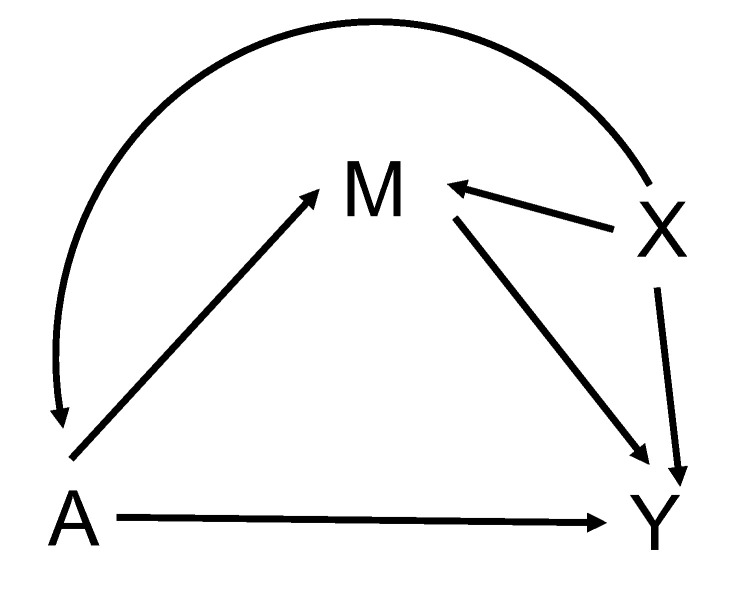
Mediation structure with a single mediator. In this model, *A* denotes the exposure variable, *M* is the mediator, *Y* is the outcome variable, and *X* is the confounder.

In this section, we briefly review the mediation model for continuous outcomes and continuous mediators proposed by VanderWeele [36]. Let *A* be the exposure, *M* be the mediator, *Y* be the outcome, and *X* be the confounder. The relationship between *A*, *M*, and *Y* is shown in Figure 2. The counterfactual variables are introduced to define the effects to be estimated and to state the assumptions needed for estimation. For example, $Y_{a}$ represents a subject’s counterfactual outcome if the exposure *A* were set at level *a*. Similarly, $M_{a}$ denotes the subject’s counterfactual mediator value if exposure were set to *a*. $Y_{am}$ denotes the counterfactual outcome value when the exposure is set to *a* and the mediator *M* is fixed at a specific value *m*.

To identify mediation effects, several assumptions must hold. First, there should be no unmeasured confounding in the exposure–outcome relationship when conditioning on *X*. This implies that all variables influencing *A* and *Y* are measured and included in *X* so that $Y_{aM_{a}}⊥A|X$. Second, there should be no unmeasured confounding in the exposure–mediator relationship when conditioning on *X*. This means that all common causes of *A* and *M* are adjusted via *X* to ensure $M_{a}⊥A|X$. Third, for the mediator–outcome relationship, it is necessary to condition on *A* and *X* so that $Y_{am}⊥M|A,X$. This ensures that no unmeasured confounders affect both *M* and *Y*. Fourth, there is no mediator–outcome confounder that is affected by the exposure. This condition ensures that there are no variables acting as confounders for the *M*–*Y* relationship that are influenced by *A* and can be expressed as $Y_{am}⊥M_{a^{*}}|X$, where $M_{a^{*}}$ is the mediator under an alternative exposure $a^{*}$.

In addition to these assumptions, consistency and positivity assumptions are also required. The consistency assumption ensures that the observed outcomes correspond to the counterfactual outcomes under the same exposure and mediator levels. The positivity assumption requires that every level of *A* and *M* has a nonzero probability for any values of *X*. Mathematically, this can be expressed as P(A=a∣X=x)>0 and P(M=m∣A=a,X=x)>0 for all a,x, and *m*.

If all the above assumptions hold, then we have

(1) $$\begin{array}{c} E\left[Y_{aM_{a^{*}}}\right]=E_{X}\left[E_{M|A=a^{*},X}\left[E\left[Y|A=a,M=m,X=x\right]\right]\right] \end{array}$$

We are interested in three mediation effects: the NDE, NIE, and the total effect (TE). The NDE measures the amount of outcome change if the exposure of an individual changes from A=a to $A=a^{*}$ with the mediator set to the level it would have taken if the exposure had been $A=a^{*}$ (i.e., $Y_{aM_{a^{*}}}-Y_{a^{*}M_{a^{*}}}$). It captures the direct effect from A→Y. The NIE is the difference in outcome for an individual with exposure A=a and a mediator set to the value it would have taken at the level of exposure A=a versus the individual whose mediator would have taken the level of exposure $A=a^{*}$ (i.e., $Y_{aM_{a}}-Y_{aM_{a^{*}}}$). It captures the indirect effect through A→M→Y. The TE is the difference in outcome for an individual having their exposure change from A=a to $A=a^{*}$ (i.e., $Y_{a}-Y_{a^{*}}$). The TE can be decomposed into the NIE and NDE:

(2) $$\begin{array}{cc} TE & =E\left[Y_{a}-Y_{a^{*}}\right]=E\left[Y_{aM_{a}}-Y_{a^{*}M_{a^{*}}}\right]=E\left[Y_{aM_{a}}-Y_{aM_{a^{*}}}\right]+E\left[Y_{aM_{a^{*}}}-Y_{a^{*}M_{a^{*}}}\right]\\ \; & =NIE+NDE \end{array}$$

If *Y* and *M* are continuous and *A* is binary, the mediation effects are modeled through the following regression equations:

(3) $$\begin{array}{cc} E\left[M|A=a,X=x\right] & =\beta_{0}+\beta_{1}a+\beta_{2}x \end{array}$$

(4) $$\begin{array}{cc} E\left[Y|A=a,M=m,X=x\right] & =\theta_{0}+\theta_{1}a+\theta_{2}m+\theta_{3}am+\theta_{4}x \end{array}$$

The NDE and NIE can be estimated with the following equations:

(5) $$\begin{array}{cc} NDE & =\left[\theta_{1}+\theta_{3}\left(\beta_{0}+\beta_{1}a^{*}+\beta_{2}x\right)\right]\left(a-a^{*}\right) \end{array}$$

(6) $$\begin{array}{cc} NIE & =\left(\theta_{2}\beta_{1}+\theta_{3}\beta_{1}a\right)\left(a-a^{*}\right) \end{array}$$

While VanderWeele’s causal mediation model provides a solid foundation for estimating mediation effects, it has several limitations that may restrict its applicability in certain contexts. One key challenge is satisfying the identifying assumptions, particularly in observational studies where unmeasured confounding could violate these assumptions and bias the results. Additionally, the model assumes linear relationships between variables, which may not hold in biomedical research datasets that are often complex and involve interactions among features. Furthermore, the model is not designed to handle high-dimensional datasets, such as those encountered in microbiome studies, where the number of mediators can vastly exceed the sample size, and interactions among mediators are prevalent. Although the challenge of satisfying identifying assumptions is a common limitation in mediation analysis and remains difficult to address, the other limitations highlight the need for advanced methods, such as Med-CNN, that can tackle non-linear relationships and high-dimensional data.

### 4.2. Proposed Neural-Network-Based Mediation Model: Med-CNN

#### 4.2.1. Framework of Med-CNN Algorithm

We introduce Med-CNN, denoted as h(), a novel deep-learning algorithm designed for mediation analysis in high-dimensional biological datasets with inherent hierarchical structures. For example, in the human microbiome, OTUs have inherent correlations within taxonomic levels and non-linear relationships between OTUs. CNNs address these challenges by leveraging their ability to model spatial dependence through convolutional layers and activation functions that capture non-linear effects. To illustrate the biological relevance of mediation analysis in this context, consider the role of dietary habits in influencing gut inflammation through changes in gut microbiome composition. Studies such as Turpin et al. have shown that Mediterranean diets rich in fiber, polyphenols, and omega-3 fatty acids promote beneficial gut microbiota, which in turn produce anti-inflammatory metabolites like short-chain fatty acids [37]. These microbiome changes mediate the relationship between diet and gut inflammation.

In the context of mediation analysis, we consider a study with *n* subjects indexed by *i*, where i=1,⋯n. Dietary habit serves as the binary exposure variable, denoted by $A_{i}$, and gut inflammation is the continuous outcome, which is denoted by $Y_{i}$. The mediators in this analysis are the high-dimensional features of the gut microbiome, which are denoted as $Z_{i}$ for the *i*-th subject. These features are organized into *K* distinct networks based on taxonomic levels and organized according to similarities observed from microbiome sequencing data. Each network *k* (k=1,⋯,K) contains $J_{k}$ microbiome features. Specifically, $Z_{kji}$ denotes the *j*-th feature in the *k*-th network for the *i*-th subject, where $j=1,...,J_{k}$. The vector of microbiome features for the *k*-th network in the *i*-th subject is represented as $Z_{ki}={\left[Z_{k1i},Z_{k2i},\cdots ,Z_{kJ_{k}i}\right]}^{T}$.

To integrate these high-dimensional features, we introduced the IMM, denoted as $h(Z_{i})$, which serves as a composite score that aggregates information across all networks. This IMM is computed through the Med-CNN model, which employs an integrated CNN model. Specifically, each microbiome network is analyzed by a network-specific CNN model designed to extract important biological features. For each network, these extracted features are condensed into a final hidden layer, which is denoted as $P_{k}$. The $P_{k}$ values from all networks are then concatenated to form a composite layer, which is used to predict the $h(Z_{i})$.

**Figure 3:**
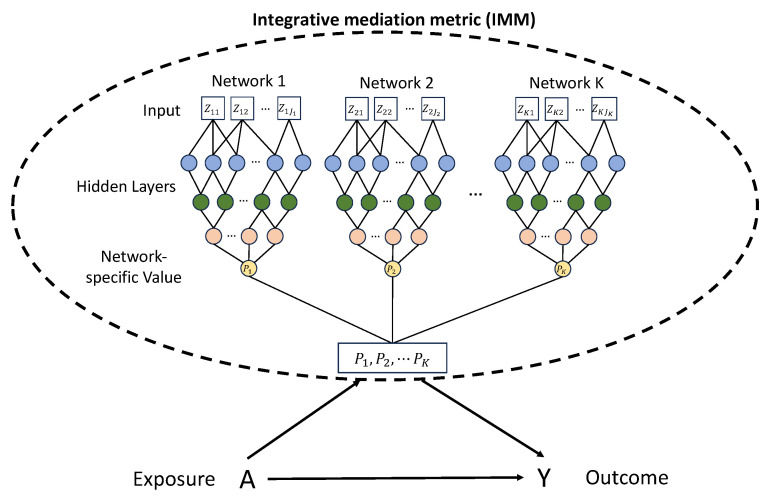
Illustration of the Med-CNN framework among *K* networks. Here, a ‘network’ is defined as a set of biologically related features organized based on their functional or structural relationships, such as OTUs clustered by phylum level or gene expressions aggregated into pathways. Each network contains $J_{k}$ features, where *k* ranges from 1 to *K*.

The Med-CNN framework is trained with an objective function that incorporates the outcome model, which ensures that the IMM is not only a summary of the biological features but also optimized to minimize the loss of the underlying mediation model. This approach ensures that the IMM is directly connected to the outcome, rather than being just an unsupervised dimensionality reduction technique. Once the IMM is computed, it is further incorporated into a single mediator model to estimate mediation parameters. Figure 3 provides an overview of our proposed algorithm.

#### 4.2.2. Network-Specific CNN Model

To enhance the efficiency of the CNN framework and effectively capture the relationship among biological features, we employed a preprocessing step suggested by Sharma et al. [29]. This approach involves reordering the input biological features based on their inter-correlations within each network. Specifically, for the $J_{k}$ biological features identified within a network, we construct a $J_{k}\times J_{k}$ correlation matrix, which is defined as

(7) $$\rho =\left[\begin{array}{ccccc} \rho_{11} & \rho_{12} & \rho_{13} & \cdots & \rho_{1J_{k}}\\ \rho_{21} & \rho_{22} & \rho_{23} & \cdots & \rho_{2J_{k}}\\ \rho_{31} & \rho_{32} & \rho_{33} & \cdots & \rho_{3J_{k}}\\ \vdots & \vdots & \vdots & \ddots & \vdots \\ \rho_{J_{k}1} & \rho_{J_{k}2} & \rho_{J_{k}3} & \cdots & \rho_{J_{k}J_{k}} \end{array}\right].$$

This matrix is then summarized into a vector $\rho_{Z}=\left\{\rho_{Z_{row_{1}}},\rho_{Z_{row_{2}}},\cdots ,\rho_{Z_{row_{J_{k}}}}\right\}$, where each element of $\rho_{Z}$ represents a composite correlation coefficient for each feature computed by

(8) $$\begin{array}{cc} \; & \rho_{row_{j}}=\sqrt[{J}]{|\rho_{j1}\left|\cdot \right|\rho_{j2}\left|\cdots \cdot \right|\rho_{jJ_{k}}|},\;\;\;\mathrm{where}\;j\in \left[1,J_{k}\right]. \end{array}$$

The vector $\rho_{Z}$ is then arranged in descending order and denoted by $\rho_{Z}*$. The input biological features are then reordered in accordance with the sequence in $\rho_{Z}*$ to preserve the inherent relationships within each network and potentially enhance the efficiency of the CNN input. This preprocessing step has been validated in TaxoNN, where correlation-based reordering improved prediction accuracy compared to alternative ordering strategies [29].

**Figure 4:**
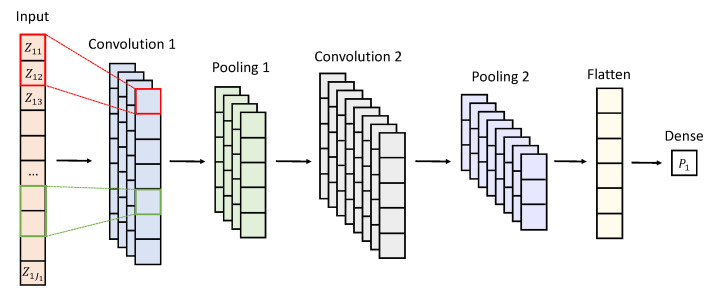
Illustration of the neural network layers for Network 1, with $P_{1}$ denoting the network-specific value associated with Network 1. All networks follow a similar architecture.

Each network-specific CNN model is structured to include two 1D convolutional layers, with each followed by a pooling layer. The initial convolutional layer contains 32 filters with a kernel size of 5 and a stride of 1. The subsequent layer contains 64 filters and maintains the same kernel size. These hyperparameters were chosen based on their demonstrated effectiveness in prior studies involving high-dimensional microbiome datasets [29]. The Rectified Linear Unit activation function was applied to both convolutional layers to introduce non-linearity [38]. The output from the final pooling layer was then flattened and processed through a dense layer with a linear activation function to generate the network-specific value. A graphical illustration of the neural network’s layer for Network 1 is shown in Figure 4.

#### 4.2.3. Loss Function and Parameter Estimation

Our algorithm employs a loss function $L\left(h(Z_{i})\right)$, inspired by Nath et al. [30], and which is formulated as

(9) $$\begin{array}{cc} L\left(h(Z_{i})\right) & =\sum_{i=1}^{n}\left({\left(y_{i}-\alpha_{0}-\alpha_{1}A_{i}-\alpha_{2}h\left(Z_{i}\right)\right)}^{2}+{\left(h\left(Z_{i}\right)-\beta_{0}-\beta_{1}A_{i}\right)}^{2}\right). \end{array}$$

This function is designed to optimize the mediation regression based on a simplified version of VanderWeele’s mediation model, with the mediation relationships expressed as

(10) $$\begin{array}{cc} \; & E\left(h\left(Z_{i}\right)|A_{i}\right)=\beta_{0}+\beta_{1}A_{i} \end{array}$$

(11) $$\begin{array}{cc} \; & E\left(Y_{i}|A_{i},Z_{i}\right)=\alpha_{0}+\alpha_{1}A_{i}+\alpha_{2}h\left(Z_{i}\right). \end{array}$$

In our Med-CNN algorithm, we utilized the $h(Z_{i})$ as the mediator in these regression models. Our model explicitly omits confounders and the interaction between exposure and mediator to reduce computational demands and demonstrate the model’s capabilities.

An iterative method, analogous to the Expectation–Maximization algorithm, was employed to fit our machine learning model and estimate the mediation parameters. This method consists of two steps. In the first step, we estimate the IMM by training the Med-CNN by assuming that the mediation parameters ($\alpha_{0}$, $\alpha_{1}$, $\alpha_{2}$, $\beta_{0}$, and $\beta_{1}$) are known. Under this assumption, the loss function is simplified as

(12) $$\begin{array}{cc} L\left(h(Z_{i})\right) & =\sum_{i=1}^{n}\left({\left(y_{i}-\alpha_{0}-\alpha_{1}A_{i}-\alpha_{2}h\left(Z_{i}\right)\right)}^{2}+{\left(h\left(Z_{i}\right)-\beta_{0}-\beta_{1}A_{i}\right)}^{2}\right)\\ \; & \propto \sum_{i=1}^{n}\left(\left(\alpha_{2}^{2}+1\right){\left(h\left(Z_{i}\right)-r_{i}\right)}^{2}\right),\mathrm{where}\;r_{i}=\frac{\left(y_{i}-\alpha_{0}-\alpha_{1}A_{i}\right)\alpha_{2}+\left(\beta_{0}+\beta_{1}A_{i}\right)}{\alpha_{2}^{2}+1}\\ \; & \propto \sum_{i=1}^{n}\left({\left(h\left(Z_{i}\right)-r_{i}\right)}^{2}\right), \end{array}$$

where $r_{i}$ is a function of known parameters, leaving $h(Z_{i})$ as the only unknown. This allows the CNN model to predict the IMM by optimizing the mediation regression and minimizing the loss function.

The second step focuses on optimizing the coefficients in the regression models (10) and (11) assuming the IMM is known. The normalized predicted IMM is incorporated into the mediation regressions to update the parameter estimates. This step enables the estimation of mediation model parameters and effects. This iterative training and updating process continues until the percentage change in each of the five parameters falls below the prespecified criteria. The convergence for each iteration is calculated using the following equation:

(13) $$Convergence=\frac{|G_{l+1}-G_{l}|}{G_{l}}\times 100\%$$

Here, *G* represents the estimated parameters at iteration *l*. Convergence is achieved when the percentage change for all mediation parameters ($\alpha_{0}$, $\alpha_{1}$, $\alpha_{2}$, $\beta_{0}$, and $\beta_{1}$) falls below the specified threshold. This convergence formula evaluates the relative improvement of the estimated parameters across iterations, avoiding scale-related issues and providing a consistent stopping criterion applicable across all parameters.

### 4.3. Simulation Studies

#### 4.3.1. Simulation Setup and Scenarios

We conducted five simulation scenarios to evaluate the performance of the proposed method. In the simulation studies, the exposure variable $A_{i}$ followed a Bernoulli distribution with a probability of 0.5. The true IMM $M_{i}$ and the outcome $Y_{i}$ were generated according to Equations (10) and (11), with error terms $\epsilon_{Mi}$ and $\epsilon_{Yi}$ following normal distributions $N\left(0,\sigma_{M}\right),N\left(0,\sigma_{Y}\right)$, respectively. Each network was modeled as a linear function of the true IMM. Network-specific biological features were incorporated as linear functions in all simulations, while non-linear relationships were additionally explored in the second and third simulation scenarios. The Med-CNN model then predicted the IMM, which is denoted ${\hat{M}}_{i}=h\left(Z_{i}\right)$. Further details of data generation can be found in Appendix A.

The first simulation scenario aimed to validate the general functionality and performance of the Med-CNN model across various null and alternative hypothesis scenarios. Specifically, we evaluated whether Med-CNN produced stable and accurate estimates of mediation effects under the following four mediation effect conditions: (1) no mediation effects ($\alpha_{2}=\beta_{1}=0$), (2) no effects of the mediator on the outcome ($\alpha_{2}=0$ but $\beta_{1}\neq 0$), (3) no effect of the exposure on the mediator $\beta_{1}=0$ but $\alpha_{2}\neq 0$, and (4) a non-zero mediation effect ($\alpha_{2}\neq \beta_{1}\neq 0$). In the non-zero mediation effect scenario, we further evaluated the influence of varying the effect size of the exposure on the mediator by setting $\beta_{1}$ to values of −5,−3,−1, 1, 3, and 5. For other model parameters, we set $\beta_{0}=2,\;,\alpha_{0}=1.5,\;\alpha_{1}=2,\;\alpha_{2}=5,\;\sigma_{M}=2,\;\sigma_{Y}=2$. The sample size was fixed at 1000, and we considered 5 networks with 100,150,120,130, and 140 biological features in each network. This setup extends TaxoNN’s 4-network design for microbiome analysis to incorporate additional biological complexity [29].

The second simulation scenario compared the performance of our proposed method with the SD-based and Reg-based approaches under the scenario of non-zero NIE. The SD-based method, developed by Huang and Pan, uses spectral decomposition to address high-dimensional mediators by transforming the correlated mediators into uncorrelated components. This transformation simplifies the estimation process by using a series of low-dimensional regression models [27]. The Reg-based method, introduced by Zhang et al., employs sure independent screening to reduce the number of potential mediators, followed by applying the minimax concave penalty method to select significant mediators [10]. We evaluated the performance of these methods under both linear and non-linear relationships between biological features and networks. Details of the feature–network relationships generation are provided in Appendix A.

In the third simulation, we assessed the performance of our method alongside the SD-based method and Reg-based method in the presence of interactions among biological features within each network under the scenario of non-zero NIE. We aimed to explore how each method handles additional complexities introduced by these interactions. To simulate these biological interactions, we selected features at intervals. Specifically, we simulated every fifth feature (e.g., 5th, 10th, 15th, etc.) to represent interacting pairs. For each pair, we modified the value of a subsequent feature by adding a term that represents the product of the interacting features’ value, which was scaled by a predefined interaction strength of 2. Further details on the generation of these interactions can be found in Appendix A.

In the fourth simulation, we aimed to determine the optimal convergence threshold for Med-CNN training under scenarios with a non-zero NIE. Convergence thresholds of 1%, 0.1%, and 0.01% were evaluated to explore the trade-off between computational efficiency and the precision of parameter estimates. These thresholds were chosen to reflect varying degrees of precision, with stricter thresholds providing finer parameter convergence at the cost of increased computational time.

Lastly, we conducted a simulation scenario to evaluate the scalability of Med-CNN with increasing sample sizes. We simulated datasets with sample sizes ranging from 500 to 1500 and assessed Med-CNN’s performance in estimating mediation effects.

For each simulation, we assessed the model’s precision by calculating the bias and the SD of the estimates for both the NIE and NDE across simulations. These metrics provide a comprehensive assessment of the accuracy and variability of the mediation estimates across simulations. SEs associated with these estimates are reported in the Appendix B.

#### 4.3.2. Model Specification and Evaluation Criteria

For the training of Med-CNN, the dataset was partitioned into a training set and a testing set with a 70/30 split. This split is a commonly used standard in deep learning to balance sufficient data for model training and adequate data for testing. Specifically, 70% of the subjects were allocated to the training set and 30% to the testing set. The simulation was conducted 100 times, with each iteration up to a maximum of 50 updates to refine the model. The updates would stop earlier if the convergence criteria were met. From each iteration, the mediation effects were estimated.

The simulations were conducted using Python 3.8.13 and R 4.2.2 on the SciNet-SOSCIP joint GPU cluster Mist server with one node allocated for each simulation [39,40]. The server was equipped with IBM Power9 cores, 256 GB of RAM, and 4 NVIDIA V100-SMX2-32GB GPUs. The computation time of each iteration of the Med-CNN algorithm is summarized in Table 4.

### 4.4. Real-Data Study

To assess the performance of our proposed method, we applied our method to vaginal microbiome data collected from a study of reproductive-age women [31]. The study contains 394 participants from North America who were recruited with informed consent from clinical sites at the University of Maryland School of Medicine and Emory University. Each participant completed the questionnaire and provided vaginal swabs. The vaginal microbiome data were obtained using pyrosequencing of barcoded 16S rRNA genes, resulting in 305 OTUs.

Our objective was to investigate whether the vaginal microbiome mediates variations in vaginal pH levels across different ethnicity groups. Four ethnicity groups (white (24.6%), black (26.4%), Hispanic (24.6%), and Asian (24.4%)) were dichotomized into white versus others. Vaginal pH levels were a continuous variable ranging from 4 to 7. For the vaginal microbiome, we classified OTUs into four networks based on their phylum-level classifications to incorporate the taxonomic hierarchy within the data. The top three phyla with the highest number of OTUs were individually selected, while the remaining phyla were aggregated into the “other” network. Specifically, the major networks for Med-CNN were identified as Firmicutes, Proteobacteria, Actinobacteria, and others. Additional details regarding the OTUs in each network can be found in Appendix C.

## Figures and Tables

**Table 1:** Results of Simulation 1, evaluating the model performance across four mediation effect scenarios: (1) no mediation effects ($\alpha_{2}=\beta_{1}=0$), (2) no effects of the mediator on the outcome ($\alpha_{2}=0$ but $\beta_{1}\neq 0$), (3) no effect of the exposure on the mediator $\beta_{1}=0$ but $\alpha_{2}\neq 0$, and (4) a non-zero mediation effect ($\alpha_{2}\neq \beta_{1}\neq 0$). In Scenario (4), we further assessed the impact of varying the effect size of $\beta_{1}$ on model performance. Reported values are derived from training datasets using a deep learning model.

		NIE			NDE		
**Scenarios**		**True Value**	**Bias**	**SD**	**True Value**	**Bias**	**SD**
Null							
(1)		0	−0.002	0.11	2	0.04	0.18
(2)		0	−0.05	1.15	2	0.09	1.15
(3)		0	−0.08	0.67	2	0.06	0.24
Alternative ($\beta_{1}$)							
	−5	−25	0.53	1.13	2	−0.53	0.80
	−3	−15	−0.16	0.78	2	0.14	0.38
(4)	−1	−5	−0.22	0.68	2	0.20	0.24
	1	5	0.09	0.70	2	−0.11	0.26
	3	15	0.38	0.89	2	−0.38	0.51
	5	25	0.56	1.74	2	−0.58	1.47

**Table 2:** Results of Simulation 2, assessing the performance of Med-CNN in comparison to SD-based and Reg-based approaches in a non-zero mediation effect scenario ($\alpha_{2}\neq \beta_{1}\neq 0$). Reported values are based on training datasets using a deep learning model.

Feature–Network		NIE			NDE		
Relationship	Method	True Value	Bias	SD	True Value	Bias	SD
Linear	Med-CNN	25	0.56	1.74	2	−0.58	1.47
	SD-based	25	0.24	0.75	2	−0.11	0.29
	Reg-based	25	12.73	4.94	2	−12.59	4.89
Non-linear	Med-CNN	25	0.17	0.73	2	−0.19	0.34
	SD-based	25	2.01	1.25	2	−1.87	1.23
	Reg-based	25	1.03	0.90	2	−0.90	0.70

**Table 3:** Results of Simulation 3, evaluating the performance of Med-CNN model in handling interactions between biological features compared to SD-based and Reg-based approaches under a non-zero mediation effect scenario. Reported values are based on results from training datasets using a deep learning model.

Feature–Network		NIE			NDE		
Relationship	Method	True Value	Bias	SD	True Value	Bias	SD
Linear	Med-CNN	25	0.42	0.81	2	−0.44	0.46
	SD-based	25	6.68	0.72	2	−6.54	0.86
	Reg-based	25	12.82	5.20	2	−12.05	5.10
Non-linear	Med-CNN	25	0.27	0.75	2	−0.29	0.39
	SD-based	25	13.27	2.39	2	−13.14	2.47
	Reg-based	25	0.81	0.98	2	−0.76	0.74

**Table 4:** Results of Simulation 4 assessing the performance of Med-CNN across three convergence thresholds: 1%, 0.1%, 0.01%.

Convergence	NIE			NDE			Computation
Thresholds	True Value	Bias	SD	True Value	Bias	SD	Time (min)
1%	25	2.23	4.32	2	−2.23	4.19	11.60
0.1%	25	0.75	2.19	2	−0.76	1.94	18.53
0.01%	25	0.56	1.74	2	−0.58	1.47	18.63

**Table 5:** Results of Simulation 5, assessing the performance of Med-CNN across different sample sizes: 500, 1000, 1500.

Sample Sizes	NIE			NDE		
	True Value	Bias	SD	True Value	Bias	SD
500	25	3.48	3.82	2	−3.46	3.68
1000	25	0.56	1.74	2	−0.58	1.47
1500	25	0.13	0.84	2	−0.26	0.67

**Table 6:** Estimated effects from the real data example.

Scenarios	Effect	Standard Error
NIE	0.06	0.001
NDE	−0.30	0.008

## Data Availability

The vaginal microbiome data analyzed during the study are from publicly accessible bacterial 16S rRNA gene sequences, which are available at the National Center for Biotechnology Information Short Read Archive (SRA022855).

## Supplementary Materials

The following supporting information can be downloaded at: https://www.mdpi.com/article/10.3390/ijms26051819/s1.

## Abbreviations

The following abbreviations are used in this manuscript:

Med-CNN	Mediation CNN
OTUs	Operational Taxonomic Units
CNNs	Convolutional Neural Networks
IMM	Integrative Mediation Metric
SD	Standard Deviation
NIE	Natural Indirect Effect
NDE	Natural Direct Effect
SD-based	Spectral Decomposition-based method
Reg-based	Regularization-based approach
SE	Standard Error
TE	Total Effect

## Appendix A. Data Generation

We generate $A_{i}$, *M*, $Y_{i}$, $P_{ki}$, $Z_{kji}$ for i=1,⋯,N as follows:

(A1) $$\begin{array}{cc} A_{i} & ∼Ber(0.5) \end{array}$$

(A2) $$\begin{array}{cc} M_{i} & =\beta_{0}+\beta_{1}A_{i}+\epsilon_{Mi} \end{array}$$

(A3) $$\begin{array}{cc} Y_{i} & =\alpha_{0}+\alpha_{1}A_{i}+\alpha_{2}M_{i}+\epsilon_{Y_{i}} \end{array}$$

(A4) $$\begin{array}{cc} P_{ki} & =\gamma_{0k}+\gamma_{1k}M_{i}+\epsilon_{Pki},\;\;\mathrm{for}\;k=1,\cdots ,K \end{array}$$

(A5) $$\begin{array}{cc} Z_{kji} & =\theta_{0kj}+\theta_{1kj}P_{ki}+\epsilon_{Zkji},\;\;\mathrm{for}\;j=1,\cdots ,J_{k} \end{array}$$

where $\epsilon_{M}∼N\left(0,2\right)$, $\epsilon_{Y}∼N\left(0,2\right)$, $\epsilon_{Pk}∼N\left(0,1\right)$, $\epsilon_{Zkj}∼N\left(0,1\right)$, $\gamma_{0k}∼N\left(0,1\right)$, $\gamma_{1k}∼Unif\left(1,5\right)$, $\theta_{0kj}∼N\left(0,1\right)$, and $\theta_{1kj}∼Unif\left(1,5\right)$.

In the second simulation, the non-linear relationship between feature–network is as follows:

(A6) $$\begin{array}{cc} Z_{kji} & =\theta_{0kj}+\theta_{1kj}P_{ki}^{2}+\theta_{2kj}P_{ki}^{3}+\epsilon_{Zkji} \end{array}$$

where $\theta_{0kj}∼Unif\left(-5,5\right)$, $\theta_{1kj}∼Unif\left(-2,2\right)$, $\theta_{2kj}∼Unif\left(-0.1,0.1\right)$, and $\epsilon_{Zkji}∼N\left(0,1\right)$.

In the third simulation, the interactions between features within the network are generated as follows:

(A7) $$Z_{kji}^{\prime }=Z_{kji}+\lambda \left(Z_{kpi}\times Z_{kqi}\right)$$

where $Z_{kji}^{\prime }$ represents the modified feature value after incorporating interaction; $Z_{kpi}$ and $Z_{kqi}$ are selected interacting features which chosen from every fifth feature within the network; and λ is a constant that defines the strength of the interaction term.

## Appendix B. Standard Errors of Simulation Results Across All Scenarios

**Table A1:** Results of Simulation 1, evaluating the model performance across four mediation effect scenarios with standard errors.

Scenarios		NIE SE	NDE SE
(1)		0.01	0.02
(2)		0.02	0.04
(3)		0.56	0.03
	−5	0.62	0.12
	−3	0.58	0.05
(4)	−1	0.57	0.03
	1	0.56	0.04
	3	0.57	0.07
	5	0.63	0.15

**Table A2:** Results of Simulation 2, assessing the performance of Med-CNN in comparison to SD-based and Reg-based approaches in a non-zero mediation effect scenario with standard errors.

Feature–Network	Relationship	NIE SE	NDE SE
Linear	Med-CNN	0.63	0.15
	SD-based	0.78	0.28
	Reg-based	5.54	5.55
Non-linear	Med-CNN	0.61	0.06
	SD-based	2.36	2.64
	Reg-based	0.87	0.58

**Table A3:** Results of Simulation 3, evaluating the performance of Med-CNN model in handling interactions between biological features compared to SD-based and Reg-based approaches under a non-zero mediation effect scenario with standard errors.

Feature–Network	Relationship	NIE SE	NDE SE
Linear	Med-CNN	0.61	0.09
	SD-based	0.61	0.70
	Reg-based	4.73	4.74
Non-linear	Med-CNN	0.61	0.07
	SD-based	2.73	2.92
	Reg-based	0.88	0.58

**Table A4:** Results of Simulation 4, assessing the performance of Med-CNN across three convergence thresholds: 1%, 0.1%, 0.01% with standard errors.

Convergence Thresholds	NIE SE	NDE SE
1%	0.62	0.23
0.1%	0.63	0.16
0.01%	0.63	0.15

**Table A5:** Results of Simulation 5, assessing the performance of Med-CNN across different sample sizes: 500, 1000, 1500 with standard errors.

Sample Sizes	NIE SE	NDE SE
500	1.25	0.51
1000	0.63	0.15
1500	0.42	0.08

## Appendix C. OTUs in Each Network

Additional details regarding the OTUs in each network can be found in the Supplementary Materials.

## Appendix D. Estimating the Standard Error of NIE and NDE by Delta Method

The mediation model is

$$\begin{array}{cc} E\left(h\left(Z_{i}\right)|A_{i}\right) & =\beta_{0}+\beta_{1}A_{i}\\ E\left(Y_{i}|A_{i},h\left(Z_{i}\right)\right) & =\alpha_{0}+\alpha_{1}A_{i}+\alpha_{2}h\left(Z_{i}\right) \end{array}$$

By the delta method, the standard error of the NIE and NDE can be estimated by

(A8) $$\begin{array}{c} SE_{NIE}=\sqrt{\Gamma_{NIE}\Sigma \Gamma_{NIE}^{T}}\left|a-a^{*}\right| \end{array}$$

(A9) $$\begin{array}{c} SE_{NDE}=\sqrt{\Gamma_{NDE}\Sigma \Gamma_{NDE}^{T}}\left|a-a^{*}\right| \end{array}$$

where the NIE and NDE are estimated by

(A10) $$\begin{array}{cc} NIE & =E\left(Y_{aM_{a}}-Y_{aM_{a^{*}}}\right)=\alpha_{2}\beta_{1}\left(a-a^{*}\right) \end{array}$$

(A11) $$\begin{array}{cc} NDE & =E\left(Y_{aM_{a}^{*}}-Y_{a^{*}M_{a^{*}}}\right)=\alpha_{1}\left(a-a^{*}\right), \end{array}$$

and $\Gamma_{NIE}$ and $\Gamma_{NDE}$ are the derivative of the NIE and NDE with respective to the αs and βs:

(A12) $$\begin{array}{cc} \Gamma_{NIE} & =\frac{\partial_{NIE}}{\partial_{\beta ,\alpha }}=\frac{\partial \left(\alpha_{2}\beta_{1}\right)}{\partial_{\beta ,\alpha }}=\left[\begin{array}{ccccc} 0 & \alpha_{2} & 0 & 0 & \beta_{1} \end{array}\right] \end{array}$$

(A13) $$\begin{array}{cc} \Gamma_{NDE} & =\frac{\partial_{NDE}}{\partial_{\beta ,\alpha }}=\frac{\partial \left(\alpha_{1}\right)}{\partial_{\beta ,\alpha }}=\left[\begin{array}{ccccc} 0 & 0 & 0 & 1 & 0 \end{array}\right]. \end{array}$$

The Σ value is the variance–covariance matrix from the mediation model:

(A14) $$\begin{array}{c} \Sigma =\left[\begin{array}{cc} \Sigma_{\beta } & 0\\ 0 & \Sigma_{\theta } \end{array}\right] \end{array}$$

(A15) $$\begin{array}{c} \Sigma_{\beta }=\left[\begin{array}{cc} \mathrm{var}\left(\beta_{0}\right) & \mathrm{cov}\left(\beta_{0},\beta_{1}\right)\\ \cdots & \mathrm{var}\left(\beta_{1}\right) \end{array}\right] \end{array}$$

(A16) $$\begin{array}{c} \Sigma_{\alpha }=\left[\begin{array}{ccc} \mathrm{var}\left(\alpha_{0}\right) & \mathrm{cov}\left(\alpha_{0},\alpha_{1}\right) & \mathrm{cov}\left(\alpha_{0},\alpha_{2}\right)\\ \cdots & \mathrm{var}\left(\alpha_{1}\right) & \mathrm{cov}\left(\alpha_{1},\alpha_{2}\right)\\ \cdots & \cdots & \mathrm{var}\left(\alpha_{2}\right) \end{array}\right]. \end{array}$$

## References

[1] Baron R.M., Kenny D.A. (1986). The moderator–mediator variable distinction in social psychological research: Conceptual, strategic, and statistical considerations. J. Personal. Soc. Psychol..

[2] Pearl J. (2022). Direct and indirect effects. Probabilistic and Causal Inference: The Works of Judea Pearl.

[3] Imai K., Keele L., Yamamoto T. (2010). Identification, inference and sensitivity analysis for causal mediation effects. Stat. Sci..

[4] VanderWeele T.J., Vansteelandt S. (2010). Odds ratios for mediation analysis for a dichotomous outcome. Am. J. Epidemiol..

[5] VanderWeele T., Vansteelandt S. (2014). Mediation analysis with multiple mediators. Epidemiol. Methods.

[6] Imai K., Yamamoto T. (2013). Identification and sensitivity analysis for multiple causal mechanisms: Revisiting evidence from framing experiments. Political Anal..

[7] Boca S.M., Sinha R., Cross A.J., Moore S.C., Sampson J.N. (2014). Testing multiple biological mediators simultaneously. Bioinformatics.

[8] Daniel R.M., De Stavola B.L., Cousens S., Vansteelandt S. (2015). Causal mediation analysis with multiple mediators. Biometrics.

[9] Jérolon A., Baglietto L., Birmelé E., Alarcon F., Perduca V. (2021). Causal mediation analysis in presence of multiple mediators uncausally related. Int. J. Biostat..

[10] Zhang H., Zheng Y., Zhang Z., Gao T., Joyce B., Yoon G., Zhang W., Schwartz J., Just A., Colicino E. (2016). Estimating and testing high-dimensional mediation effects in epigenetic studies. Bioinformatics.

[11] Zhao Y., Luo X. (2022). Pathway Lasso: Pathway estimation and selection with high-dimensional mediators. Stat. Its Interface.

[12] Chén O.Y., Crainiceanu C., Ogburn E.L., Caffo B.S., Wager T.D., Lindquist M.A. (2018). High-dimensional multivariate mediation with application to neuroimaging data. Biostatistics.

[13] Latorre M., Krishnareddy S., Freedberg D.E. (2015). Microbiome as mediator: Do systemic infections start in the gut?. World J. Gastroenterol..

[14] Schulz M.D., Atay Ç., Heringer J., Romrig F.K., Schwitalla S., Aydin B., Ziegler P.K., Varga J., Reindl W., Pommerenke C. (2014). High-fat-diet-mediated dysbiosis promotes intestinal carcinogenesis independently of obesity. Nature.

[15] Taur Y., Pamer E.G. (2016). Microbiome mediation of infections in the cancer setting. Genome Med..

[16] Lutz S.M., Hokanson J.E. (2014). Genetic influences on smoking and clinical disease. Understanding behavioral and biological pathways with mediation analysis. Ann. Am. Thorac. Soc..

[17] Teng M.S., Hsu L.A., Wu S., Sun Y.C., Juan S.H., Ko Y.L. (2015). Association of CDH13 genotypes/haplotypes with circulating adiponectin levels, metabolic syndrome, and related metabolic phenotypes: The role of the suppression effect. PLoS ONE.

[18] Wu G.D., Chen J., Hoffmann C., Bittinger K., Chen Y.Y., Keilbaugh S.A., Bewtra M., Knights D., Walters W.A., Knight R. (2011). Linking long-term dietary patterns with gut microbial enterotypes. Science.

[19] Lewis J.D., Chen E.Z., Baldassano R.N., Otley A.R., Griffiths A.M., Lee D., Bittinger K., Bailey A., Friedman E.S., Hoffmann C. (2015). Inflammation, antibiotics, and diet as environmental stressors of the gut microbiome in pediatric Crohn’s disease. Cell Host Microbe.

[20] Kurilshikov A., Wijmenga C., Fu J., Zhernakova A. (2017). Host genetics and gut microbiome: Challenges and perspectives. Trends Immunol..

[21] Kim D., Zeng M.Y., Núñez G. (2017). The interplay between host immune cells and gut microbiota in chronic inflammatory diseases. Exp. Mol. Med..

[22] Turnbaugh P.J., Hamady M., Yatsunenko T., Cantarel B.L., Duncan A., Ley R.E., Sogin M.L., Jones W.J., Roe B.A., Affourtit J.P. (2009). A core gut microbiome in obese and lean twins. Nature.

[23] Qin J., Li Y., Cai Z., Li S., Zhu J., Zhang F., Liang S., Zhang W., Guan Y., Shen D. (2012). A metagenome-wide association study of gut microbiota in type 2 diabetes. Nature.

[24] Koeth R.A., Wang Z., Levison B.S., Buffa J.A., Org E., Sheehy B.T., Britt E.B., Fu X., Wu Y., Li L. (2013). Intestinal microbiota metabolism of L-carnitine, a nutrient in red meat, promotes atherosclerosis. Nat. Med..

[25] Soskic B., Cano-Gamez E., Smyth D.J., Ambridge K., Ke Z., Matte J.C., Bossini-Castillo L., Kaplanis J., Ramirez-Navarro L., Lorenc A. (2022). Immune disease risk variants regulate gene expression dynamics during CD4^+^ T cell activation. Nat. Genet..

[26] Britt E.C., John S.V., Locasale J.W., Fan J. (2020). Metabolic regulation of epigenetic remodeling in immune cells. Curr. Opin. Biotechnol..

[27] Huang Y.T., Pan W.C. (2016). Hypothesis test of mediation effect in causal mediation model with high-dimensional continuous mediators. Biometrics.

[28] Sharma D., Xu W. (2023). ReGeNNe: Genetic pathway-based deep neural network using canonical correlation regularizer for disease prediction. Bioinformatics.

[29] Sharma D., Paterson A.D., Xu W. (2020). TaxoNN: Ensemble of neural networks on stratified microbiome data for disease prediction. Bioinformatics.

[30] Nath T., Caffo B., Wager T., Lindquist M.A. (2023). A machine learning based approach towards high-dimensional mediation analysis. NeuroImage.

[31] Ravel J., Gajer P., Abdo Z., Schneider G.M., Koenig S.S., McCulle S.L., Karlebach S., Gorle R., Russell J., Tacket C.O. (2011). Vaginal microbiome of reproductive-age women. Proc. Natl. Acad. Sci. USA.

[32] Shishpal P., Patel V., Singh D., Bhor V.M. (2021). pH Stress Mediated Alteration in Protein Composition and Reduction in Cytotoxic Potential of Gardnerella vaginalis Membrane Vesicles. Front. Microbiol..

[33] Serrano M.G., Parikh H.I., Brooks J.P., Edwards D.J., Arodz T.J., Edupuganti L., Huang B., Girerd P.H., Bokhari Y.A., Bradley S.P. (2019). Racioethnic diversity in the dynamics of the vaginal microbiome during pregnancy. Nat. Med..

[34] Fettweis J.M., Brooks J.P., Serrano M.G., Sheth N.U., Girerd P.H., Edwards D.J., Strauss J.F., Jefferson K.K., Buck G.A., Consortium V.M. (2014). Differences in vaginal microbiome in African American women versus women of European ancestry. Microbiology.

[35] Beamer M.A., Austin M.N., Avolia H.A., Meyn L.A., Bunge K.E., Hillier S.L. (2017). Bacterial species colonizing the vagina of healthy women are not associated with race. Anaerobe.

[36] Valeri L., VanderWeele T.J. (2013). Mediation analysis allowing for exposure–mediator interactions and causal interpretation: Theoretical assumptions and implementation with SAS and SPSS macros. Psychol. Methods.

[37] Turpin W., Dong M., Sasson G., Garay J.A.R., Espin-Garcia O., Lee S.H., Neustaeter A., Smith M.I., Leibovitzh H., Guttman D.S. (2022). Mediterranean-like dietary pattern associations with gut microbiome composition and subclinical gastrointestinal inflammation. Gastroenterology.

[38] Nair V., Hinton G.E. Rectified linear units improve restricted boltzmann machines. Proceedings of the 27th International Conference on Machine Learning (ICML-10).

[39] Python Core Team (2019). Python: A Dynamic, Open Source Programming Language.

[40] R Core Team (2021). R: A Language and Environment for Statistical Computing.

